# Characterization and phylogenetic analysis of the complete mitochondrial genome of *Gomphus clavatus* (pers.) Gray, 1821

**DOI:** 10.1080/23802359.2026.2742635

**Published:** 2026-10-14

**Authors:** Bowen Zan, Xiao Zou, Liangju Li, Bangmeng Zhou, Lixiu Yan, Guangsen Tong, Luming Yang, Xiangbo Xu

**Affiliations:** ^a^ School of Culinary and Food Science Engineering, Sichuan Tourism University, Chengdu, P.R. China; ^b^ Chongqing Academy of Metrology and Quality Inspection, Chongqing, P.R. China

**Keywords:** Mitochondrial genome, *Gomphus clavatus*, phylogenetic analysis, Basidiomycota, Gomphaceae

## Abstract

The mitochondrial genome of *G. clavatus* was sequenced, assembled, and annotated in this study. The complete mitochondrial genome is a circular molecule of 85,207 bp, with a GC content of 28.50%. The overall nucleotide composition is asymmetric, with A (35.30%) and T (36.21%) being more prevalent than G (14.81%) and C (13.69%). It encodes a total of 62 genes, including 15 core protein-coding genes (PCGs), 2 rRNA genes (*rns* and *rnl*), 25 tRNA genes, 19 intron-encoded open reading frames (ORFs), and 1 free-standing ORF. A total of 16 introns were identified within the genome. A phylogenetic tree was constructed using Bayesian inference (BI) and maximum likelihood (ML) based on a joint dataset of 15 mitochondrial core PCGs. The analysis robustly places *G. clavatus* in a clade with *Ramaria ichnusensis* (PP847338), indicating a close genetic relationship between these species. This study provides the first complete mitogenome resource for *G. clavatus*, offering crucial molecular data for its taxonomic placement and enriching fungal mitochondrial genome databases.

## 1. Introduction

*Gomphus clavatus* (Pers.) Gray, 1821, commonly known as the pig’s ear fungus, is a distinctive macrofungus belonging to the family Gomphaceae within the order Gomphales (Giachini et al. 2010; Fan et al. 2023). This species forms ectomycorrhizal associations with coniferous trees, particularly spruce and fir, playing a critical role in nutrient cycling and forest ecosystem health (Geng et al. 2023). *G. clavatus* is also valued as an edible fungus with a pleasant taste and thick flesh, making it of economic interest in several regions including Southwest China (Makropoulou et al. 2012; Geng et al. 2022; Liu et al. 2022). Despite its ecological and economic significance, the taxonomic placement of *Gomphus* has long been controversial due to morphological convergence with related genera within the cantharelloid-gomphoid fungi (Vidal et al. 2023). The genus *Gomphus* currently comprises several species. However, the lack of comprehensive molecular data has hindered a robust understanding of its evolutionary relationships and divergence timelines (Fan et al. 2023).

To characterize the mitochondrial genome of *G. clavatus* and resolve its phylogenetic position, this study sequenced, assembled, and annotated its complete mitochondrial genome for the first time. A multi-gene phylogenetic tree was constructed to accurately place this species within the Basidiomycota. This study provides new data resources for fungal mitochondrial evolution research and advances our understanding of the Gomphales phylogenetic system.

## 2. Materials and methods

### 2.1. Sample collection and identification

Fresh specimens of *G. clavatus* were collected from coniferous forests in Longquan mountain, Chengdu, Sichuan Province, China (30.55 N, 104.30 E) (Figure 1). The samples were immediately placed on ice packs for transport to the laboratory. Morphological identification was performed based on macroscopic characteristics. For molecular identification, the internal transcribed spacer (ITS) region was amplified using primers ITS1 and ITS4 under standard PCR conditions, and the amplicon was Sanger-sequenced (PZ945750). BLAST search against the GenBank database showed 99.56% identity with *G. clavatus* voucher HKAS 133652 (PV715619), and a Neighbor‑Joining tree based on ITS sequences (Figure 3b) further confirmed the species identification. A voucher specimen was deposited at the Culture Collection Center of Sichuan Tourism University under the accession number Gcl06 (contact person: Bowen Zan; email: zbwa1996@163.com).

**Figure 1.**
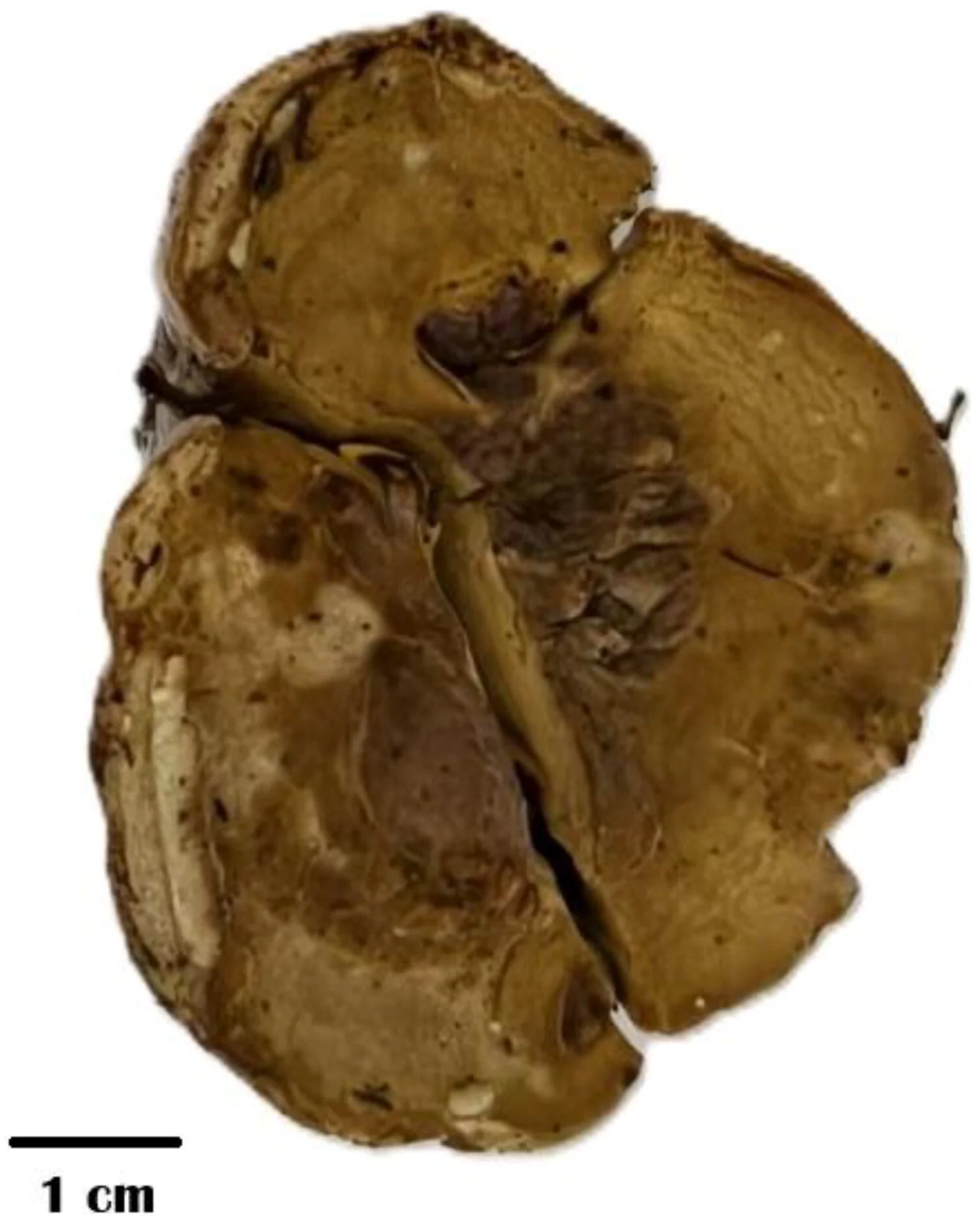
Basidiocarps of *gomphus clavatus* collected from natural habitat, showing typical features: funnel-shaped to fan-shaped pileus with a wrinkled or merulioid hymenium, and a robust stipe. Photo was taken by Bowen Zan.

### 2.2. Mitochondrial genome sequencing, assembly, and annotation

Total genomic DNA was extracted from the fruiting bodies of *G. clavatus* using a commercial fungal DNA extraction kit (Omega Bio-Tek, Norcross, GA, USA). A sequencing library was constructed following the manufacturer’s protocol. Paired-end (2 × 150 bp) sequencing was performed on an Illumina platform. Raw reads were subjected to quality control: low-quality sequences and adapter contaminants were removed using ngsShoRT (Chen et al. 2014) and AdapterRemoval v2 (Schubert et al. 2016), respectively. *De novo* assembly of the mitochondrial genome was conducted using NOVOPlasty v4.3.3 with k-mer of 29 (Dierckxsens et al. 2017). The assembled mitochondrial genome was initially annotated using MFannot (Valach et al. 2014) and MITOS (Bernt et al. 2013) webservers. Protein-coding genes (PCGs) and open reading frames (ORFs) were predicted or verified using the NCBI ORF Finder (Coordinators 2017). Functional annotation of PCGs/ORFs was performed by BLASTP searches against the NCBI non-redundant protein database (Bleasby and Wootton 1990). Exon-intron boundaries were detected using exonerate v2.2 (Slater and Birney 2005). Intron classification was performed using RNAweasel (version 1.0) and the Rfam database (version 14.9) for structural motif identification. Transfer RNA (tRNA) genes were identified and verified with tRNAscan-SE v1.3.1 (Lowe and Chan 2016). A circular map of the mitochondrial genome was generated using PMGmap (http://www.1kmpg.cn/pmgmap) (Zhang et al., 2024).

### 2.3. Phylogenetic analysis

Phylogenetic reconstruction was carried out according to previously described methods. *Hannaella oryzae* (MH732752) was selected as the outgroup (Li et al. 2021). Fifteen conserved mitochondrial protein-coding genes (*atp6*, *atp8*, *atp9*, *cob*, *cox1*, *cox2*, *cox3*, *nad1*, *nad2*, *nad3*, *nad4*, *nad4L*, *nad5*, *nad6*, and *rps3*) were extracted from the mitochondrial genome of *G. clavatus* and 13 other Basidiomycota species. Intron regions, if present, were excluded. Each gene was individually aligned using MAFFT v7.037 (Katoh et al. 2019). The aligned sequences were then concatenated into a single dataset using SequenceMatrix v1.7.8 (Vaidya et al. 2011). A partition homogeneity test was performed to assess potential conflicts among genes. The optimal partitioning scheme and corresponding nucleotide substitution models for the concatenated dataset were determined using PartitionFinder 2.1.1 (Lanfear et al. 2017). Phylogenetic trees were inferred using both Bayesian Inference (BI) and Maximum Likelihood (ML) methods. BI analysis was conducted with MrBayes v3.2.6 (Ronquist et al. 2012). ML analysis was performed with RAxML v8.0.0 (Stamatakis 2014), conducting 10,000 rapid bootstrap replicates to assess nodal support.

## 3. Results

Sequencing of the *G. clavatus* Gcl06 mitochondrial genome achieved an average coverage depth of 457.83× (Figure. S1). The complete mitochondrial genome was assembled into a circular molecule of 85,207 bp, exhibiting a GC content of 28.50%. The overall nucleotide composition was asymmetric, with A (35.30%) and T (36.21%) being more prevalent than G (14.81%) and C (13.69%). Annotation of the genome identified a total of 62 genes. This complement includes 15 core protein-coding genes (PCGs) (*atp6*, *atp8*, *atp9*, *cob*, *cox1*, *cox2*, *cox3*, *nad1*, *nad2*, *nad3*, *nad4*, *nad4L*, *nad5*, *nad6*, and *rps3*), 2 ribosomal RNA (rRNA) genes (*rns* and *rnl*), 25 transfer RNA (tRNA) genes, 19 intron-encoded open reading frames (ORFs) (Figure. S2), and 1 free-standing ORF of unknown function (Figure 2).

**Figure 2.**
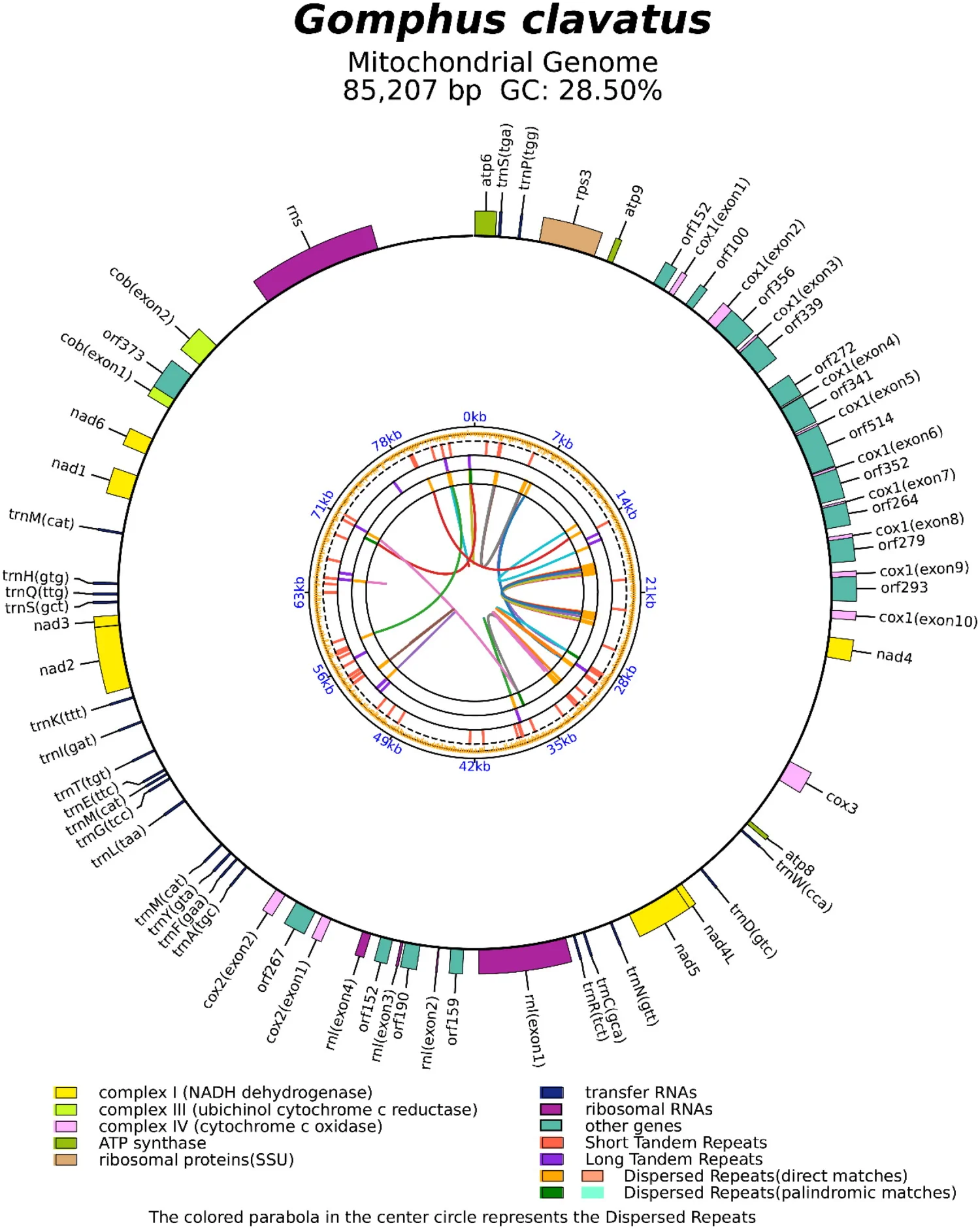
The circular mitochondrial genome map of *gomphus clavatus*. Different color blocks represent different genes. The colored parabola in the center circle represents the dispersed repeats.

A total of 16 introns were identified within the mitochondrial genome. These introns were classified into six groups: 7 Group IB introns, 4 Group IA introns, 2 Group ID introns, 1 Group IC2 intron, 1 Group II intron, and 1 Group I (derived) intron. The intronic ORFs predominantly encode LAGLIDADG and GIY-YIG homing endonucleases, with a minority encoding hypothetical proteins. These endonucleases are believed to facilitate the lateral transfer of genetic material and play significant roles in the evolution of mitochondrial genomes.

Phylogenetic analyses based on the concatenated sequences of the 15 core mitochondrial PCGs were performed using both Bayesian Inference (BI) and Maximum Likelihood (ML) methods (Figure 3). The resulting trees from both approaches were topologically congruent and showed strong nodal support. The analysis robustly placed *G. clavatus* Gcl06 (PZ559951) within a clade alongside *Ramaria ichnusensis* (PP847338), indicating a close phylogenetic relationship between these species. This placement is consistent with the known taxonomic affinity within the Gomphales and provides molecular evidence supporting the evolutionary relationships within this group.

**Figure 3.**
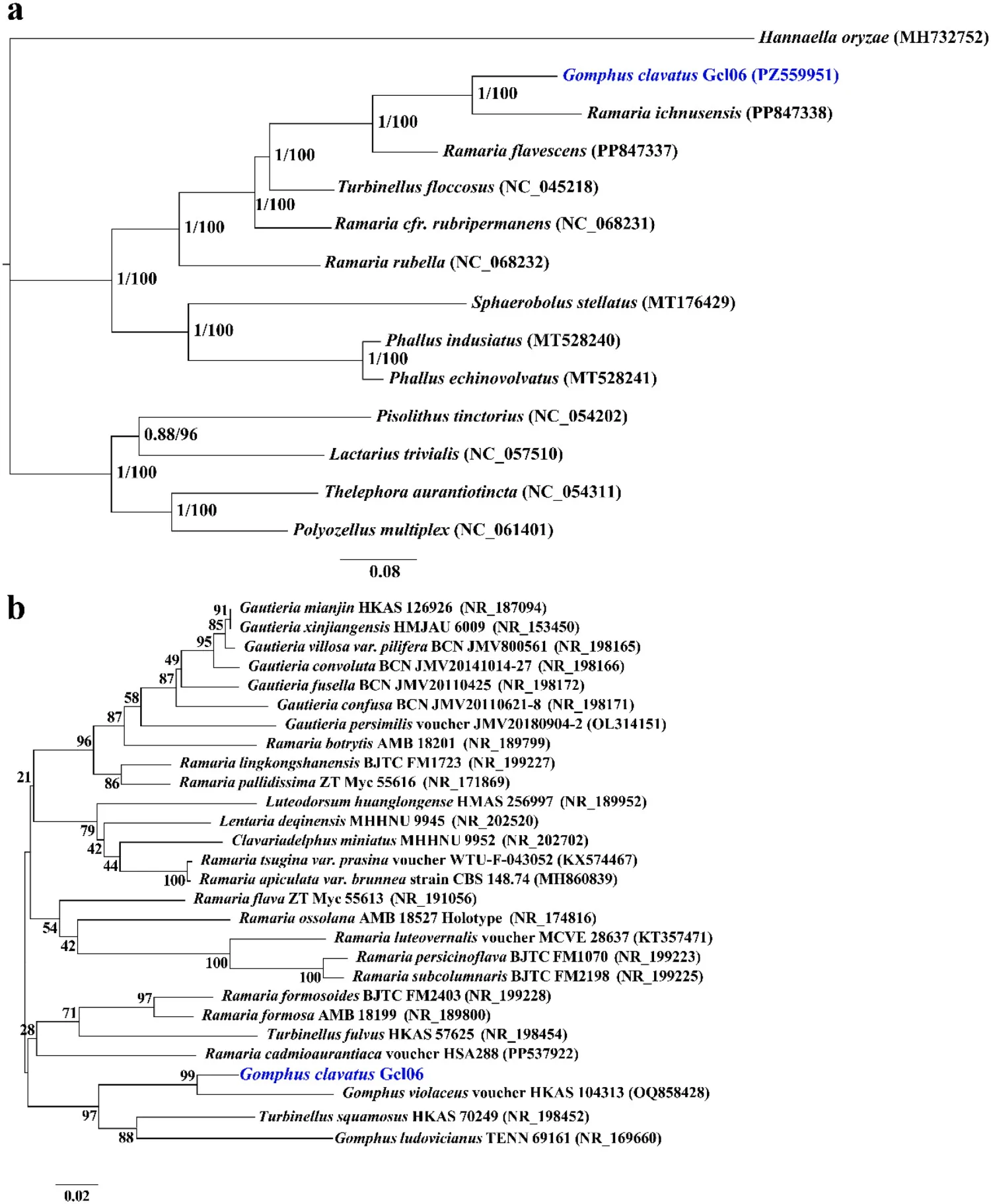
Bayesian inference and maximum likelihood tree generated using 15 concatenated mitochondrial protein-coding genes (*atp6*, *atp8*, *atp9*, *cob*, *cox1*, *cox2*, *cox3*, *nad1*, *nad2*, *nad3*, *nad4*, *nad4L*, *nad5*, *nad6*, and *rps3*) from *gomphus clavatus* Gcl06 and other fungal species (a). The support values are Bayesian posterior probabilities (BPPs) and bootstrap values (BSs). Phylogenetic tree based on Neighbor-Joining method using ITS sequences (b). *Hannaella oryzae* (MH732752) was used as the outgroup (Li et al. 2021). the accession number information of the sequence is as follows: *Turbinellus floccosus* (NC_045218) (Cheng et al. 2021), *ramaria cfr. rubripermanens* (NC_068231) (Li et al. 2022), *ramaria rubella* (NC_068232) (Li et al. 2022), *lactarius trivialis* (NC_057510) (Shao et al. 2020), *sphaerobolus stellatus* (MT176429) (Ye et al. 2020), *phallus indusiatus* (MT528240) (Chen et al. 2020), *thelephora aurantiotincta* (NC_054311) (Chen et al. 2021), *polyozellus multiplex* (NC_061401) (Liu et al. 2022), *ramaria ichnusensis* (PP847338) (Wang et al. 2025), *ramaria flavescens* (PP847337) (Wang et al. 2025), *phallus echinovolvatus* (MT528241) (Chen et al. 2020), *pisolithus tinctorius* (NC_054202) (Wu et al. 2021).

## 4. Discussion and conclusion

Mitochondrial genomes have become indispensable molecular tools for resolving phylogenetic relationships and understanding evolutionary histories across fungal lineages (Li et al. 2019; Zardoya 2020; Zhang et al., 2024). Prior to this study, the mitochondrial genome of *G. clavatus* remained uncharacterized, limiting the application of mitogenomic data in clarifying its taxonomy and evolutionary placement among other macrofungi. Our work fills this knowledge gap by presenting the first complete mitochondrial genome for *G. clavatus*. The mitogenome of *G. clavatus* displays a relatively large size of 85,207 bp, which is larger than that of the closely related *Ramaria ichnusensis* (61,851 bp) and *Ramaria flavescens* (81,282 bp) (Liu et al. 2024). This size variation is primarily attributed to differences in intergenic spacer length and intron content (Zubaer et al. 2018; Jia et al. 2025). The low GC content (28.50%) is consistent with the typical base composition of fungal mitochondrial genomes and reflects the AT-rich nature of these organelles (Song et al. 2022; Yu and Liang 2022).

A notable feature of the *G. clavatus* mitogenome is the presence of 16 introns distributed across various protein-coding genes, particularly within *cox1* and *rnl*. The diversity of intron groups—including Group IA, IB, IC2, ID, II, and Group I (derived)—highlights the dynamic nature of fungal mitochondrial genomes (Sinn and Barrett 2020; Nieuwenhuis et al. 2023). The predominance of LAGLIDADG and GIY-YIG homing endonucleases encoded by these intronic ORFs suggests that intron mobility and horizontal transfer have played significant roles in shaping the genome architecture of *G. clavatus* (Wu and Hao 2014).

## Supplementary Material

Supplementary figure.docx

## Data Availability

The genome sequence data that support the findings of this study are openly available in the NCBI GenBank under accession no. PZ559951. The associated BioProject, SRA, and Bio-Sample numbers are PRJNA1480416, SRR39247205 and SAMN61028588, respectively.
