# Supplementary material for: Characterization and phylogenetic analysis of the complete mitochondrial genome of Gomphus clavatus (pers.) Gray, 1821
Source: Mitochondrial DNA B Resour. 2026 Oct 14;11(11):1294–9. doi: 10.1080/23802359.2026.2742635 (PMC13670273; doi:10.1080/23802359.2026.2742635)
Supplement: Supplementary file 1 [file TMDN_A_2742635_SM2193.docx]

**Supplementary Figure S1** Sequencing depth and coverage map of the *Gomphus clavatus* mitochondrial genome. The x-axis represents the genomic position (bp), and the y-axis represents the sequencing depth (×).

**Supplementary Figure S2** Cis-splicing gene structures in the *Gomphus clavatus* mitochondrial genome. Exons are shown as colored boxes, and exon numbers and positions are indicated. The genes shown are *cob, cox1, cox2*, and *rnl*. No trans-splicing genes were detected in this mitogenome.
